# Role of Long Noncoding RNA HOTAIR in the Growth and Apoptosis of Osteosarcoma Cell MG-63

**DOI:** 10.1155/2016/5757641

**Published:** 2016-08-31

**Authors:** Hua Zheng, Jing Min

**Affiliations:** Department of Orthopedics, Yongchuan Hospital, Chongqing Medical University, Chongqing, China

## Abstract

This study investigated the function of HOTAIR in the growth and apoptosis of OS MG-63 cell line* in vitro* and further clarified its mechanism. The expression levels of HOTAIR in OS MG-63 cell line and normal osteoblast hFOB1.19 cell line were determined by RT-PCR, respectively. The growth and apoptosis of MG-63 cells* in vitro* were investigated by MTT assay and flow cytometry assay after HOTAIR was knocked down with retroviral vector construction. And the expression levels of cell growth and apoptosis related factors TGF-*β*, p53, Bcl-2, and TNF-*α* were determined to clarify the mechanism. We found that HOTAIR was highly expressed in osteosarcoma MG-63 cell line compared with normal osteoblast hFOB1.19 cell line. The proliferation rate was lower and the apoptosis rate was higher significantly in shHOTAIR MG-63 cells than those in EV MG-63 cells. TGF-*β* and Bcl-2 were downregulated significantly when HOTAIR was knocked down. p53 and TNF-*α* were upregulated significantly when HOTAIR was knocked down. These results indicated that HOTAIR functioned as a carcinogenic lncRNA, which could promote the proliferation and inhibit the apoptosis of MG-63 cells* in vitro*. HOTAIR could be a potential target for the treatment of osteosarcoma.

## 1. Introduction

Osteosarcoma, which derives from primitive bone-forming mesenchymal cells, is a primary malignant tumor of the skeleton. It was characterized by the direct formation of immature bone or osteoid tissue by the tumor cells. Although it accounts for less than 0.5% of all types of cancer, OS is the most frequent primary malignancy of the bone and occurs mainly in adolescents and young adults [1]. Generally, OS cases mostly occur in the long tubular bones which account for 80% to 90% and rarely affected the axial skeleton. Patients with OS suffer from severe pain and soft tissue swelling. Development of sudden and severe pain may lead to gross pathologic fracture, which is an uncommon finding in adult patients. But up to 15% of paediatric patients present a pathological fracture [2]. Although the mortality and the survival of OS have been greatly improved, the mortality of OS still ranks the third (8.9%) in all childhood and adolescent cancer deaths behind leukemias (25.5%) and brain and other nervous-system neoplasms (25.0%). Mankin et al. reported that the overall 5-year survival rate for OS was 68% without a significant gender difference based on 648 patients treated for OS at Massachusetts General Hospital by the orthopedic oncology group in 2004 [3, 4]. Thus, studies on the treatment and the pathogenesis of OS have always been hotspots in recent decades.

Long noncoding RNAs (lncRNAs) are a group of transcribed RNA consisting of more than 200 nucleotides and generally poorly conserved. They are noncoding RNAs, and they can regulate gene expression by different mechanisms which still remain unclear [5, 6]. The role of lncRNAs is very different from miRNAs as miRNAs mainly play roles in inhibiting gene expression posttranscriptionally while lncRNAs play important roles in epigenetic regulation, transcriptional control, posttranscriptional regulation, and molecular scaffolding via different mechanisms [7]. Studies have shown that lncRNAs play an important role in X-chromosome inactivation, genomic imprinting, subcellular structural organization, telomere and centromere organization, and nuclear trafficking [7–10]. Moreover, studies have indicated that the dysregulation of lncRNAs is associated with various human diseases, including autoimmune diseases, neurological disorders, coronary artery diseases, and cancer [11–13].

HOTAIR, one of the well-studied lncRNAs, was localized at human chromosome 12q13 within the antisense strand of the HOXC gene cluster [14]. Since it was discovered by Rinn et al. in 2007, several studies have shown that HOTAIR is a powerful predictor of invasion and metastasis, cell proliferation, pathological development, or drug resistance in various tumors such as non-small cell lung cancer, melanoma, and gastric cancer [15–18]. For OS, HOTAIR was only reported such that overexpression of HOTAIR could promote tumor growth and metastasis in human osteosarcoma [19].

In this study, we aimed to detect the different expression of HOTAIR in OS cell line and normal osteoblast cell line, investigate the effects of HOTAIR knockdown on growth and apoptosis of OS cells* in vitro*, and further clarify the mechanism of HOTAIR in OS.

## 2. Materials and Methods

### 2.1. Cell Culture

Human osteosarcoma MG-63 and osteoblast hFOB1.19 cell lines were purchased from the Type Culture Collection of Chinese Academy of Sciences (Shanghai, China). MG-63 cells were cultured in MEM/EBSS (Hyclone, Logan, UT, USA) supplemented with 10% heat-inactivated fetal bovine serum (FBS, Hyclone), 50 U/mL penicillin, and 50 mg/mL streptomycin in a humidified incubator with 5% CO_2_ at 37°C. The hFOB1.19 cells were cultured with DMEM/F12 (v/v: 1 : 1, Hyclone) supplemented with 10% FBS and 0.3 mg/mL G418 (Sigma, St. Louis, MO, USA) under the same conditions.

### 2.2. Total RNA Extraction and HOTAIR Quantitative Real-Time PCR

Total RNA was isolated and purified from MG-63 and hFOB1.19 cells by the RNeasy Mini Kit (Qiagen, Inc.). And cDNA was synthesized from the total RNA by reverse transcription with PrimeScript® 1st strand cDNA Synthesis Kit (TaKaRa Bio, Shiga, Japan). Sequences of all the primers are shown in the following list (Primer Sequence for PCR). The expression of HOTAIR was quantified by fluorescence-based RT-PCR amplification.

*Primer Sequence for PCR*

*Sequence (5*′*-3*′)

 TGF-*β*-Forward: 5′-GCTGTGAAGCCTTGAGAGTAATGG-3′
 TGF-*β*-Reverse: 5′-TTCCTGTTGACTGAGTTGCGATAA-3′
 p53-Forward: 5′-TACTCCCCTGCCCTCAACAAGA-3′
 p53-Reverse: 5′-CGCTATCTGAGCAGCGCTCATG-3′
 TNFa-Forward: 5′-CCTGTCTCTTCCTACCCAACC-3′
 TNFa-Reverse: 5′-GCAGGAGTGTCCGTGTCTTC-3′
 Bcl-2-Forward: 5′-ATGTGTGTGGAGAGCGTCAA-3′
 Bcl-2-Reverse: 5′-ACAGTTCCACAAAGGCATCC-3′
 HOTAIR-Forward: 5′-GGGTGTTGGTCTGTGGAACT-3′
 HOTAIR-Reverse: 5′-CAGTGG-GGAACTCTGACTCG-3′
 GAPDH-Forward: 5′-CTTTGGTATCGTGGAAGGACTC-3′
 GAPDH-Reverse: 5′-GTAGAGGCAGGGATGATGTTCT-3′

### 2.3. HOTAIR Retroviral Vector Construction and RNA Interference

To knock down HOTAIR in MG-63 cells, Knockout*™* RNAi Systems (Clontech Laboratories Inc., CA, USA) were used according to the manufacturer's protocol. Briefly, shRNA sequence targeted HOTAIR was designed and shown in Primer Sequence for PCR in Section 2.2 [20]. The complementary shRNA oligonucleotides were annealed and ligated into the pSIREN vectors (shHOTAIR). Then, Plat-A cells were transfected with shHOTAIR or pSIREN vector [21], and MG-63 cells were infected with the recombinant retroviruses and selected with puromycin. Each* in vitro* experiment was performed in triplicate.

### 2.4. Cell Proliferation Assay

1 × 10^4^ cells were seeded per well in 96 well plates in normal cell growth media. To investigate the proliferation of transfected MG-63 cells, the 3-(4,5-dimethylthiazol-2-yl)-2,5-diphenyltetrazolium bromide (MTT) assay was performed using a Cell Proliferation Kit I (GE Healthcare Life Sciences, NJ) according to the manufacturer's protocol. The absorbance at 570 nm was determined by VersaMax (Molecular Devices, CA) to estimate MTT-formazan production after 24, 48, and 72 hours of incubation to evaluate the proliferation rate. The index was evaluated at 48 and 72 hours normalized to that at 24 hours.

### 2.5. Apoptosis Rate Determination* In Vitro*

The transfected cells were fixed with 2.5% glutaraldehyde for 30 min. Annexin V-FITC and PI staining flow cytometry was used to determine apoptosis rates by determining the relative amount of Annexin V-FITC positive and PI negative cells. Unstained cells and cells stained with Annexin V-FITC or PI alone were used as controls.

### 2.6. Protein Extraction and Western Blotting

The total protein was extracted from collected MG-63 cells and separated with 10% SDS-PAGE. Then the gel was transferred to a PVDF membrane (Solvay Chemicals, Belgium) and blocked for 1 h. The rabbit anti-TGF-*β*, p53, Bcl-2, and TNF-*α* antibody and goat anti-rabbit IgG antibody were used as primary and secondary antibody at dilution of 1 : 1000 and 1 : 10000, respectively. Signal was visualized with the ECL kit (Amersham International, Amersham, UK). Image J software (NIH, Bethesda, MD, USA) was used to compare the gray values between the proteins of interest and the internal control protein, as well as between the phosphorylated protein and the total protein.

### 2.7. RT-PCR Analysis

The expression levels of TGF-*β*, p53, Bcl-2, and TNF-*α* mRNA in transfected MG-63 cells were assessed by RT-PCR. Total RNA was extracted from cells using the Trizol method, after which cDNA was synthesized from the RNA by reverse transcription. PCR amplification was performed to allow for fluorescence-based quantitation of the gene expression. PCR reaction volumes were 10 *μ*L and composed of cDNA (1 *μ*L), primers (0.2 *μ*L each), 2 × Premix Ex Taq (5 *μ*L), and H_2_O (3.6 *μ*L). The primer sequences used were listed in Primer Sequence for PCR in Section 2.2.

## 3. Results

### 3.1. The Differential Expression of HOTAIR in MG-63 and hFOB1.19 Cells

**Figure 1:**
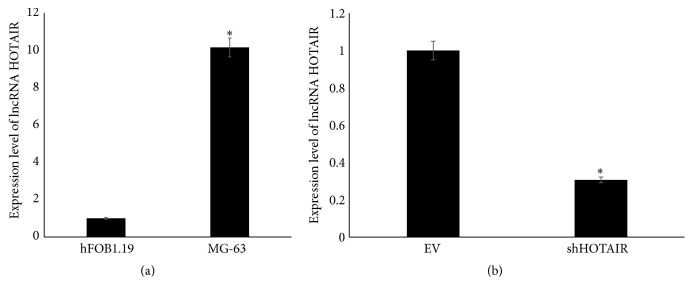
The expression level of lncRNA HOTAIR determined by RT-PCR. (a) The expression level of lncRNA HOTAIR in osteoblast cell line hFOB1.19 and osteosarcoma cell line. (b) The expression level of lncRNA HOTAIR in MG-63 transfected with empty vectors (EV) and shHOTAIR. ^*∗*^ *P* < 0.05.

The expressions of lncRNA HOTAIR in human osteosarcoma MG-63 and osteoblast hFOB1.19 cell lines were estimated and shown in Figure 1(a). HOTAIR was highly expressed in osteosarcoma MG-63 cell line compared with normal osteoblast hFOB1.19 cell line. To investigate the function of HOTAIR in osteosarcoma cells, the HOTAIR was knocked down in MG-63 cells using retroviral vectors transfection. As shown in Figure 1(b), the shHOTAIR MG-63 cells expressed significantly lower level of HOTAIR than that in MG-63 cells transfected with empty vectors.

### 3.2. The HOTAIR Was Associated with the Proliferation and Apoptosis of MG-63 Cells

**Figure 2:**
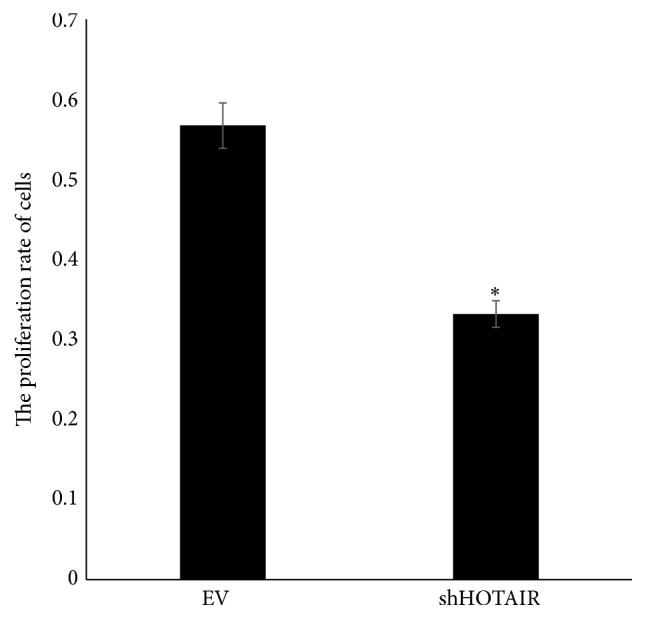
The proliferation rate of MG-63 transfected with empty vectors (EV) and shHOTAIR. ^*∗*^ *P* < 0.05.

**Figure 3:**
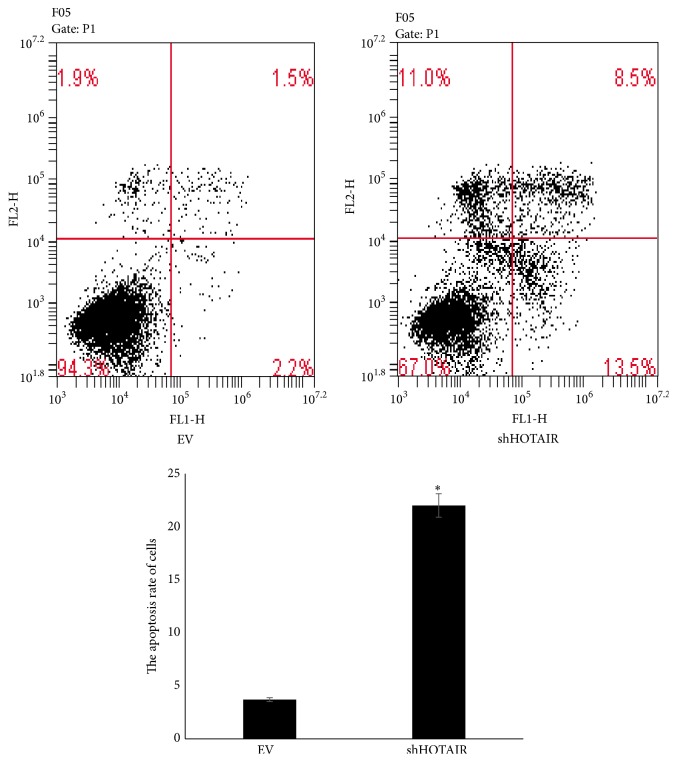
The apoptosis rate of MG-63 transfected with empty vectors (EV) and shHOTAIR. ^*∗*^ *P* < 0.05.

The proliferation and apoptosis of MG-63 cells after transfection were determined and shown in Figures 2 and 3. The proliferation rate was lower and the apoptosis rate was higher significantly in shHOTAIR MG-63 cells than those in EV MG-63 cells. These results indicated that HOTAIR could promote the proliferation and inhibit the apoptosis of MG-63 cells as a carcinogenic lncRNA.

### 3.3. Expression Level of Tumor Related Factors

**Figure 4:**
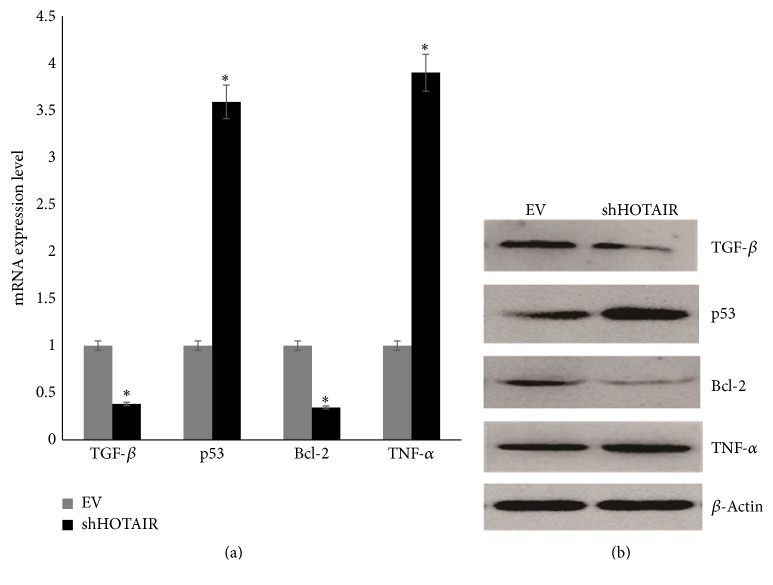
The expression level of tumor related factors in MG-63 transfected with empty vectors (EV) and shHOTAIR. (a) The expression level of mRNAs. (b) The expression level of proteins. ^*∗*^ *P* < 0.05.

Expression levels of TGF-*β*, p53, Bcl-2, and TNF-*α* mRNAs and proteins in infected MG-63 cells were determined by RT-PCR and western blotting, which were shown in Figure 4. TGF-*β* and Bcl-2 were downregulated significantly when HOTAIR was knocked down. p53 and TNF-*α* were upregulated significantly when HOTAIR was knocked down.

## 4. Discussion

In this study, major findings were that HOTAIR was highly expressed in OS cell line MG-63 compared with normal osteoblast cell line hFOB1.19, the proliferation rate was lower, and the apoptosis rate was higher significantly when HOTAIR was knocked down in MG-63 cells, and tumor related factors TGF-*β*, p53, Bcl-2, and TNF-*α* were differently expressed in MG-63 cells when HOTAIR was knocked down. HOTAIR, one of the most commonly high-regulated lncRNAs in tumor cells, could promote the proliferation and inhibit the apoptosis as an oncogenic lncRNA.

lncRNAs are commonly transcribed in the genome and play critical roles in tumor progress because of their various functions in posttranscriptional, transcriptional, and epigenetic mechanisms of gene regulation. Studies have shown that lncRNAs are abnormally expressed in various cancers and play both oncogenic and tumor suppressive roles in the development of cancer [5]. For example, Steroid Receptor RNA Activator (SRA) is a potential biomarker of steroid-dependent tumors and highly expressed in various cancers, including human uterus, ovary, and breast cancer [22]. Metastasis-Associated Lung Adenocarcinoma Transcript 1 (MALAT1) is widely expressed in normal human tissues [23, 24] and is upregulated in various human cancers, including breast, prostate, colon, liver, and uterus cancers [25–28]. While Growth Arrest-Specific 5 (GAS5) is a tumor suppressor lncRNA and is significantly downregulated in breast cancers, and GAS5 can induce cell apoptosis directly or indirectly in cancer cell lines [29]. HOTAIR is the first discovered tumor related lncRNA and is upregulated in breast, hepatocellular, colorectal, pancreatic, and other cancers in recent years [30–32]. Most existing studies mainly focus on the cancer metastasis, poor survival time, and poor prognosis. In our study, we first investigated the association between HOTAIR and OS and further clarified the effects of HOTAIR on the proliferation and apoptosis of OS cells MG-63* in vitro*. Similar results were obtained such that HOTAIR was upregulated in OS cells as an oncogenic lncRNA, which could promote the proliferation and inhibit the apoptosis of MG-63 cells.

p53 gene is the most prominent tumor suppressor gene encoding the cellular tumor antigen p53 (Tp53). It is a pivotal tumor suppressor that induces apoptosis and blocks cell cycle [33]. B-cell lymphoma-2 (bcl-2) is an inner mitochondrial membrane protein, which can block programmed cell death, inhibit apoptosis, and participate in the survival of cancer cells [34]. Many studies have proved that p53 can induce apoptosis through inhibiting bcl-2 proteins and they all function in the p53 pathway [35]. The p53 pathway is well studied in the context of regulating cell cycle and apoptosis, and emerging evidences in recent years suggest that several lncRNAs such as MEG3, MALAT1, LOC285194, and PANDA are involved in p53 pathway [6]. In this study, we detected the expression level of p53 and bcl-2 after silencing HOTAIR and found that the level of p53 was upregulated significantly while bcl-2 was downregulated. These results indicated that HOTAIR was associated in the p53-mediated apoptosis pathway in OS MG-63 cells. However, the direct target of HOTAIR in apoptosis pathway still needs further studies. TGF-*β* is a member of TGF-*β* superfamily and mainly regulates the cell growth and differentiation processes which has been reported in various cancers.* In vitro* and* in vivo* studies have shown that TGF-*β* can regulate bone formation by inducing osteoblast and osteoclast proliferation [36] and high expression level was observed in osteosarcoma cells [37]. TNF-*α* is a potent cytokine produced primarily by activated macrophages and reported to inhibit the proliferation and differentiation in osteosarcoma cells* in vitro* [38]. In our study, TGF-*β* was downregulated after silencing HOTAIR whereas TNF-*α* was upregulated in MG-63 cells. These results indicated that HOTAIR could induce the proliferation of OS MG-63 cells although the integrated mechanism still needed further investigation.

Above all, HOTAIR is associated with the tumor tumorigenesis and development of OS and can serve as a potential treatment target clinically in future.

## 5. Conclusions

HOTAIR can promote the proliferation and inhibit the apoptosis of MG-63 cells* in vitro* and can be a potential target for the treatment of osteosarcoma.
